# Depletion-of-Battery Attack: Specificity, Modelling and Analysis

**DOI:** 10.3390/s18061849

**Published:** 2018-06-06

**Authors:** Vladimir Shakhov, Insoo Koo

**Affiliations:** 1 Automobile/Ship Electronics Convergence Center, University of Ulsan, Ulsan 680-749, Korea; shakhov@mail.ulsan.ac.kr; 2 The School of Electrical Engineering, University of Ulsan, Ulsan 680-749, Korea

**Keywords:** wireless sensor networks, security, depletion-of-battery attack

## Abstract

The emerging Internet of Things (IoT) has great potential; however, the societal costs of the IoT can outweigh its benefits. To unlock IoT potential, there needs to be improvement in the security of IoT applications. There are several standardization initiatives for sensor networks, which eventually converge with the Internet of Things. As sensor-based applications are deployed, security emerges as an essential requirement. One of the critical issues of wireless sensor technology is limited sensor resources, including sensor batteries. This creates a vulnerability to battery-exhausting attacks. Rapid exhaustion of sensor battery power is not only explained by intrusions, but can also be due to random failure of embedded sensor protocols. Thus, most wireless sensor applications, without tools to defend against rash battery exhausting, would be unable to function during prescribed times. In this paper, we consider a special type of threat, in which the harm is malicious depletion of sensor battery power. In contrast to the traditional denial-of-service attack, quality of service under the considered attack is not necessarily degraded. Moreover, the quality of service can increase up to the moment of the sensor set crashes. We argue that this is a distinguishing type of attack. Hence, the application of a traditional defense mechanism against this threat is not always possible. Therefore, effective methods should be developed to counter the threat. We first discuss the feasibility of rash depletion of battery power. Next, we propose a model for evaluation of energy consumption when under attack. Finally, a technique to counter the attack is discussed.

## 1. Introduction

The technology of wireless sensor systems has evolved over the last decade from where these systems were designed in a technology-dependent manner to a stage where some broad conceptual understanding of issues now exists. Significant progress in the area of micro-electro-mechanical technologies has enabled the development of inexpensive sensor nodes that communicate wirelessly. Wireless sensor networks (WSNs) can be used in a wide range of applications, such as air pollution monitoring [1], landslide detection [2], military applications and tracking [3], healthcare [4,5,6,7], and smart homes [8,9]. There are several projects on, and standardization initiatives for, sensor networks, which may eventually converge with the Internet of Things (IoT). For example, European Union projects of Internet of Things Architecture (IoT-A) have been addressing the challenges of IoT solutions development from a WSNs perspective [10].

As sensors-based applications are deployed, security emerges as an essential requirement. Future IoT threats will disable home security systems, flood fields, and disrupt hospitals. However, wireless sensor network survivability and reliability are low. The reasons are as follows: Sensor resources are limited. Usually, mobile terminal facilities do not allow application of an efficient defense scheme against intrusions. Wireless sensor technologies are relatively new and so, the corresponding defense tools are poor. Wireless sensors use unreliable channels. In wireless communications, the signal transmission is disturbed by noise. A sensor has to contact other sensors, which facilitates the spread of infection. Thus, it is very important to investigate potential attacks against wireless sensors and IoT systems. It is necessary to improve the security taxonomy, which can help to develop practical intrusion detection and attack mitigation systems. It is required to develop mathematical models for quantitative assessment of a safety level.

There are many sources for material on security issues in WSNs. Surveys of attacks against WSNs can be found [11,12,13,14]. In particular, the taxonomy of denial-of-service (DoS) attacks in WSNs was described [15]. According to these papers, attacks against sensors can be classified into attacks on the physical, medium access control (MAC), network, transportation, and application layers. A summary of typical DoS attacks on sensor networks and possible defense techniques is as follows. An approach based on spread-spectrum and a lower duty cycle is used against the jamming attack; tamper-proofing and special key management schemes are used against the tampering attack; an error correcting code is used against link layer attacks; rate limitation is used against any type of resource exhaustion; authentication, encryption, and probing are used against manipulation of routing information, selective forwarding attacks, and Sybil attacks. Both signature-based analysis and anomaly-based analysis are wide applied for DoS attacks detection in WSNs. In some cases, it is highly successful. However, traditional intrusion detection systems cannot deal with battery-depletion attacks [16]. A WSNs node is vulnerable to battery exhaustion for the following reasons. The cost of sensors has to be low; it is a market requirement. However, the cost of batteries tends to increase in proportion to their capacity. Hence, the capacity of a WSN node battery is low. In recent papers, power-exhausting attacks have been considered [17,18,19,20,21]. The authors considered these attacks as a special type of DoS attack, as well. However, the situation introduced in this paper has not been considered. Quality of service under a battery depletion attack is not necessarily degraded. Moreover, quality of service can increase, right up to the moment some critical set of sensors crashes. This gives rise to a novel type of attack that is not DoS.

Let us assume the following scenario. Malware in an infected sensor forces the sensor transmitter to essentially increase signal power. Sensor energy consumption is increased and leads to quick battery exhaustion. Radio communications is usually among the highest energy drains on WSN nodes. At the same time, the signal-to-noise ratio is improved. Hence, the quality of signal reception is improved. The sensor transmission range is increased. Hence, packet latency can be reduced. System throughput is increased right up to the crash, which happens when a group of sensors fails and the network becomes disconnected. The attack effect looks like physical destruction. According to the International Telecommunications Union definition, DoS attacks focus on preventing legitimate users of a service from using the service. In this case, the goal of the attack is not fast service degradation. Moreover, the quality of service can be temporarily improved. Therefore, it is not a DoS attack. The target of the attack is the equipment (more exactly, the sensor battery), and the goal of attack is to disable the battery. The attack tools can be similar to a DoS situation. It can be malware or relaxed jamming. Therefore, it is not a physical attack. Here, by the term “physical attack”, we mean that an attacker commits unauthorized physical actions to completely disable a sensor node (sensor node destroying, damaging of some key component of sensor equipment, thievery). Thus, we get a special type of attack. We name it the depletion-of-battery (DoB) attack.

This paper is organized as follows. In the next two sections, we discuss the related works and the feasibility of rash depletion of battery power. Next, we describe a model for sensor battery behavior when there is no attack. We then modify this model, taking into account the specifics of a DoB attack experienced due to packets flood. Next, we consider the DoB attack itself, which is caused by excessive power in the transmit signal. Thus, we propose theoretical tools for the evaluation of energy consumption under DoB attacks. A counteracting technique against the attack is discussed as well. Performance analysis and a brief conclusion finalize the paper.

## 2. Related Works

Most researchers of WSNs security note potential threats caused by the low capacity of sensor battery. Generally, authors consider some DoS attacks and mention that the fast exhaustion of sensor energy is one of the possible effects of attacks, along with others. An example of this situation is the wormhole attack. This not only wastes communication bandwidth but also makes network nodes consume the additional energy [22]. The other example is the clone attack (nodes replication attack). Generally, here the intruder goal is to overhear the traffic, inject false data into the system, and revoke legitimate nodes. However, the energy depletion of nodes can be a concomitant effect [23].

The Vampire attack [20] is one of the first attempts to define a new class of DoS attack related to the sensor battery power depletion. The authors consider two routing layer battery depletion attacks: the carousel attack and the stretch attack. In the carousel attack, a malicious node composes packets with purposely introduced routing loops. It is assumed that a sender of a packet defines the path the packet takes through the network. The attack exploits the limited verification of message headers at forwarding nodes, allowing a single packet to repeatedly traverse the same set of nodes. The stretch attack targets source routing protocols as well. It constructs artificially long packet paths, which potentially include every node in the network. The authors evaluate the vulnerabilities of existing protocols to Vampire attacks and provide an approach to reduce the attack damage by verifying that packets consistently make progress toward their destinations. Some slight modifications of this defense method for the same two attacks can be found in the papers [21,24,25]. The authors of Vampire attack mentioned that the attack differs from traditional DoS. In this case, a defense strategy against traditional DoS does not protect against intelligent adversaries who use a small number of packets or do not originate packets at all. However, it is still a DoS attack. Obviously, the Vampire attack increases packet latency, and further the packets loss rate can be potentially increased. Thus, the quality of service degrades. Hence, traditional intrusion detection systems based on QoS degradation monitoring can be applied for the Vampire attack detection.

In the paper [26], authors considered a novel type of attacks on the energy system of a ubiquitous sensor network. The attack is based on the spurious flows generation. The authors experimentally demonstrated that the system lifetime can be reduced seven times and the intrusion effect depends more on the spurious flow intensity than on the average speed of mobile nodes.

The denial-of-sleep attack tries to keep the sensor nodes awake to consume more energy. In previous papers, it is being seen as a special type of DoS attack [17,19,27,28,29]. An intruder can broadcast a fake preamble in the sender-initiated MAC protocols. The adjacent nodes wake up, hear the fake preamble, and stay away to receive and process spoofed data from the intruder (B-MAC). The attack keeps the receivers awake as long as it exhausts the battery of sensors. An intruder can cheat the attacked sender to establish a communication session. The sender will start to send the data to the intruder but the intruder will never replay by the ACK packet (X-MAC). Therefore, the victim node does not complete data transmitting and exhausts the battery rapidly. In these scenarios, the packet delivery ratio degrades. It is also possible to organize the denial-of-sleep attack, which temporarily improves the packet latency and the packets delivery ratio. For example, an intruder can intercept and faster retransmit a flow of legal packets. However, this facility has not been properly investigated.

The battery exhaustion effect can be strengthened by data encryption. The intruder can send the encrypted spoofed data to attacked node. Before the victim identifies that the data is spoofed, the victim consumes more energy to receive and decrypt data. The threats for traditional wireless sensor networks may also occur in networks with more powerful sensor motes. Even in wireless multimedia sensor networks, an intruder may rapidly drain the energy of nodes [30]. It has been shown that mobile devices supporting IEEE 802.11 and IEEE 802.15.1 standards are vulnerable to battery exhaustion attacks [31]. Authors remark that this is DoS attack. It violates the overall operation of the device. Generally, sensors are deployed in unattended and hostile environments. Therefore, an intruder can easily receive a physical access to a sensor and extract all the stored keys [32]. The recent encryption schemes use a source-destination path key to protect data transmitted over the route instead of using multiple pairwise shared keys to repeatedly perform encryption and decryption over every link [33]. Thus, a big part of the computational and energy expenses is delegated to the sink. It improves the energy efficiency of intermediate sensors. However, it makes a system vulnerable to flooding attack.

In the paper [34] a malware based battery depletion attack is considered. The authors focus on the strategy of malware behavior and optimize the rate of network nodes infection. The infective state is modeled by one state of the four-state Markov chain. Moreover, the energy depletion time is assumed to be exponentially distributed at time. Therefore, it is assumed that the residual battery charge does not depend on time. It is not applicable for limited capacity of sensor battery. Similar simplified assumptions have been used in our previous paper [18], when the energy exhausting DoS attack has been considered. These simple models are convenient for a general consideration of defense strategies. However, it cannot be applied for comprehensive analysis of concrete attacks. In the present paper, we provide more detailed and complicated models.

Our previous papers [35,36] presented for the first time the idea that DoB is a novel type of attack that is not necessary DoS. In the present paper, we provide the next generation of research in this direction. We essentially improve the background information and totally revise the system model and survivability metrics.

## 3. DoB Feasibility and Features

DoB can be caused by deliberate action or a random combination of circumstances. A random failure of embedded sensor protocols can lead to the scenario of DoB without malware. If a routing algorithm is not energy efficient, remote sensors can be connected by direct links without intermediate nodes. It leads to latency reduction. However, WSN lifetime becomes very short.

The considered attack can be organized by relaxed jamming. If a sensor cannot adaptively change transmission power, then the intrusion is equivalent to the well-known DoS attack on WSNs called jamming. Jamming can disrupt wireless connections and can occur either unintentionally (in the form of interference) or through collisions. A jamming attack is usually effective, since expensive hardware is not required in order to launch one. It can be implemented by medium listening and broadcasting on the same frequency band as the sensors, so it can lead to significant effects with small incurred costs for the attacker. If a sensor can counteract noise by increasing transmission power, then the object of the attack becomes the battery of the sensor, wherein the signal quality and transmission range can be increased. The same effect can be caused by malware [16]. Malware can force a sensor to increase transmission power. In addition, malware can block sensors close to sink, so some other node has to increase its communication power without being able to use intermediate link to sink.

It is generally assumed that the sensor duty cycle contains a sleep mode. The need to wait for an active slot increases the latency in cases where asynchronous WSN sensors use preamble-based MAC protocols for connection setup and data transmission. It takes a random amount of time, and increases packet delay. The sleep mode can be blocked by malware. If the sleep mode is blocked, packet delay will be reduced. Moreover, increasing node throughput leads to a packet loss rate reduction. Hence, the QoS is improved. However, sensors quickly exhaust their batteries. The details of denial-of-sleep attacks and their impacts on MAC protocols (S-MAC, T-MAC, B-MAC, and G-MAC) are described in [17]. The authors mentioned that an attacked network can maintain throughput and latency similar to that of the network when it is not under attack.

Alternative methods for DoB organization can be offered as well. For example, an intruder can organize an infinite session with an attacked node. Vulnerabilities of MAC protocols can be used for frequent wake-up. Implementation of DoB attacks based on excessive transmissions, accompanied with increasing packets latency, is described in [20]. Thus, there are two types of DoB. Attacks of one type lead to QoS degradation and can be considered as the DoS attack, whereas others may improve QoS.

**Figure 1:**
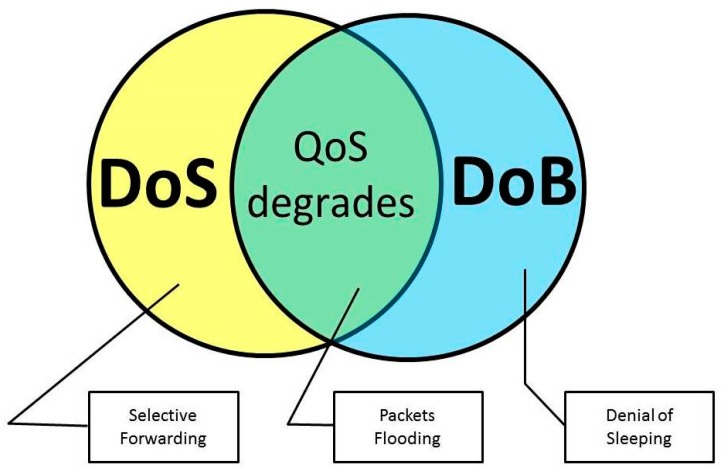
Venn diagram representation of the denial-of-service (DoS) and depletion-of-battery (DoB) attacks interrelation.

Figure 1 shows how DoS and DoB attacks interrelate. The yellow circle represents DoS attacks. Typical examples include Selective Forwarding. Some research [11,37] on the sensor network threats showed that the total number of transmissions is reduced under this attack. Hence, the total energy consumption is generally reduced. In total, Selective Forwarding can improve QoS parameters such as delay and loss rate. However, quality of information degrades; therefore, it is a DoS attack. The blue circle represents DoB attacks. The denial-of-sleep attack can represent the DoB class, when taking into consideration the arguments mentioned above. Another representative of this type of intrusion is DoB caused by excessive power in the transmit signal. The intersection of DoS and DoB is not empty. There is a set of attacks that are common to both DoS and DoB. For example, attacks based on packets flooding (see, for example [38]) lead to buffer overflow in sensors, the low packet delivery ratio, the increased packet latency as well as rash battery exhausting. In the next section, we consider in detail the DoB attack caused by excessive packets transmissions, accompanied with QoS degradation, and the DoB attack caused by excessive transmission power, with possible QoS improvement. The provided results can be adapted for other DoB attacks.

## 4. DoB Analysis

In this section, we present a theoretical framework for modeling and analyzing DoB attacks. The proposed models are based on continuous time Markov chains (CTMC). This technique is generally used when the various performance metrics of WSNs are calculated. For example, these metrics include the effectiveness of battery recovery [39], characteristics of Rayleigh fading channels [40], the average number of successful transmissions completed by WSN nodes [41], and the effectiveness of protection against timing attacks [42]. Here, we consider the impact of a DoB attack, as well as the sensor battery capacity, on the system lifetime. Approaches to WSN lifetime estimation are discussed, as well.

### 4.1. System Model

Let us consider a node in a WSN that transmits its own generated data and retransmits data of other sensors. We first discuss the sensor behavior under normal conditions, i.e., any attacks are absent. Next, the proposed model will be easily modified for intrusion cases. The transmitted packet stream is assumed to be Poisson with an average transmission rate of *λ* packets per time unit. Let $e_{0}$ be the energy needed to handle one packet. Assume the energy consumption for packet transmitting is much higher than the energy consumption for other activities (packet receiving, idle listening, monitoring, data collection). Thus, without loss of generality, $e_{0}$ is the energy consumption for transmitting one packet. If the current capacity of sensor battery is less than $e_{0}$ then the packet transmission has failed. Thus, it makes no sense to consider the energy consumption per bit in this work.

Let C be the charge capacity of a sensor battery. The total number of packets transmitted by a sensor, $N_{0}$, can be estimated as follows:

(1) $$N_{0}=C/e_{0}.$$

After further consideration, $N_{0}$ is rounded to the nearest integer.

Let the discrete random variable N(t) be the sensor lifetime at time *t* in terms of a number of transmissions. In other words, the residual battery charge at time *t* is enough for transmission of N(t) packets, but not enough for transmission of N(t)+1 packets. For our purposes, it is convenient to model the sensor behavior by the following Markov process:

{N(t), t≥0},

where $N\left(0\right)=N_{0}$. The process takes non-negative integer values. If N(t)=0, then the sensor battery is completely depleted, and the sensor is faulty. Here, *λ* is the rate at which packet transmission occurs. Let us denote the state probabilities as follows:

$$P_{k}\left(t\right)=P\left[N\left(t\right)=k\right],\;k=0,\;1,\;\ldots ,\;N_{0}.$$

It is clear that for a given *t*

$$\underset{k}{\sum }P_{k}\left(t\right)=1.$$

This process is known as the pure death process. The state probabilities are described by the following differential equations (for example, see [43])

$$\frac{dP_{k}\left(t\right)}{dt}=-\lambda \;P_{k}\left(t\right)+\lambda \;P_{k+1}\left(t\right),\;0<k<N_{0},\;\frac{dP_{N_{0}}\left(t\right)}{dt}=-\lambda \;P_{N_{0}}\left(t\right),\;\frac{dP_{0}\left(t\right)}{dt}=\lambda \;P_{0}\left(t\right),$$

with known solution

$$P_{k}\left(t\right)=\frac{{\left(\lambda t\right)}^{N_{0}-k}}{\left(N_{0}-k\right)!}\;e^{-\lambda t},\;0<k\leq N_{0}.$$

Therefore,

$$P_{0}\left(t\right)=1-e^{-\lambda t}\overset{N_{0}}{\underset{k=1}{\sum }}\frac{{\left(\lambda t\right)}^{N_{0}-k}}{\left(N_{0}-k\right)!}=1-e^{-\lambda t}\overset{N_{0}-1}{\underset{k=0}{\sum }}\frac{{\left(\lambda t\right)}^{k}}{k!}.$$

The function $P_{0}\left(t\right)$ is the probability of the sensor being faulty at time *t*. This function is useful for sensor survivability estimation. Let *T* be the sensor lifetime. It is a random variable with the cumulative distribution function (CDF) $F_{T}\left(t\right)$. By CDF definition, $F_{T}\left(t\right)$ equals the probability of the event T≤t. Hence,

$$F_{T}\left(t\right)=\mathbb{P}\left(T\leq t\right)=1-\mathbb{P}\left(T>t\right)=P_{0}\left(t\right).$$

Thus, *T* is an $N_{0}$-stage Erlang random variable. Hence, the expected sensor lifetime (also known as *mean time to failure* or MTTF) can be estimated as follows:

$$\mathrm{MTTF}=\frac{N_{0}}{\lambda }.$$

It is a commonly used survivability metric [42,44]. MTTF provides the mean time it takes for the sensor to reach the failure state for a given initial state of the sensor. This result can also be derived from the fact that process N(t) spends an exponentially distributed time in each of its states.

In WSNs investigations, researchers often consider the lifetime of the whole system. At that point, the WSN lifetime is generally defined as the time duration until the first sensor node is out of battery power [45,46,47,48,49,50,51,52]. The corresponding authors remark that the loss of a sensor quickly adds extra loads to its neighboring sensors. It leads to a rapid collapse of the network. Moreover, in some cases the network gets partitioned when the first node dies. It is reasonable to assume that critical sensor nodes are attacked first. In view of the above, we consider the following situation. Let a network contains *m* critical sensors. The failure of any critical sensor leads to the failure of whole system. Assume the sensor lifetimes, denoted by $T_{1},T_{2},\ldots ,T_{m},$ are mutually independent random variables. Therefore, the system lifetime τ is also a random variable and

$$\tau =\min\;\left\{T_{1},T_{2},\ldots ,T_{m}\right\}.$$

We get:

$$\mathbb{P}\left(\tau >t\right)=\mathbb{P}\left(T_{j}>t\;\forall j\in \bar{1,m}\right)=\overset{m}{\underset{j=1}{\prod }}\left(1-F_{T_{j}}\left(t\right)\right).$$

Therefore, the CDF of τ is defined as follows:

$$F_{\tau }\left(t\right)=1-\mathbb{P}\left(\tau >t\right).$$

If all *m* sensors are in the same condition, such that $T_{j}\;\left(1\leq j\leq m\right)$ are identically distributed variables with CDF $F_{T}\left(t\right)$, then:

$$F_{\tau }\left(t\right)=1-{\left(1-F_{T}\left(t\right)\right)}^{m}=1-e^{-\lambda m\;t}{\left(\overset{N_{0}-1}{\underset{k=0}{\sum }}\frac{{\left(\lambda \;t\right)}^{k}}{k!}\right)}^{m}.$$

In applications, it is often required to support the desired system lifetime. Let us consider the probability of the event “the system lifetime exceeds the given threshold.” For a given threshold *h* we get:

$$\mathbb{P}\left(\tau >h\right)=1-F_{\tau }\left(h\right).$$

Thus, the desired probability is defined as follows:

$$\mathbb{P}\left(\tau >h\right)=e^{-\lambda m\;t}{\left(\overset{N_{0}-1}{\underset{k=0}{\sum }}\frac{{\left(\lambda \;t\right)}^{k}}{k!}\right)}^{m}.$$

This result can be generalized. Now, for a given m, let us introduce the *system survivability function* (SSF) of some nonnegative real variables h,λ, and a nonnegative integer variable y:

$$ℋ\left(h,\lambda ,y\right):{\mathbb{R}}_{\geq 0}\times {\mathbb{R}}_{\geq 0}\times \mathbb{N}\to {\mathbb{R}}_{\left[0,1\right]}$$

as follows:

$$ℋ\left(h,\lambda ,y\right)=e^{-\lambda \;m\;h}{\left(\sum_{k=0}^{y-1}\frac{{\left(\lambda \;h\right)}^{k}}{k!}\right)}^{m}.$$

This function gives the probability that a network has not failed within time *h*. Note that ℋ(h,λ,y) is the monotonically decreasing function of *h*,

$$ℋ\left(0,\lambda ,y\right)=1,\;\underset{h\to \infty }{\lim}ℋ\left(h,\lambda ,y\right)=0.$$

We offer to use the function ℋ(h,λ,y) as an alternative survivability metric of WSNs. If DoB is absent, then the value of y is given by formula (1), and we get $ℋ\left(h,\lambda ,N_{0}\right)$. Under a DoB attack, both y and *λ* can be changed. Below, MTTF and SSF are used for DoB threat estimation.

### 4.2. DoB Based on Excessive Packets

We now consider the case where a DoB attack is organized by excessive packet generation. An intruder can generate spoofed packets, retransmit legal packets multiple times, use an inefficient routing path, or use other ways to increase the offered load on the sensors. In this case, the traffic intensity essentially grows, and obviously, QoS is degraded. The novel traffic rate is:

$$\lambda^{*}=\left(\lambda_{A}+\lambda )p_{A}+\lambda (1-p_{A}\right)=\lambda_{A}p_{A}+\lambda .$$

**Figure 2:**
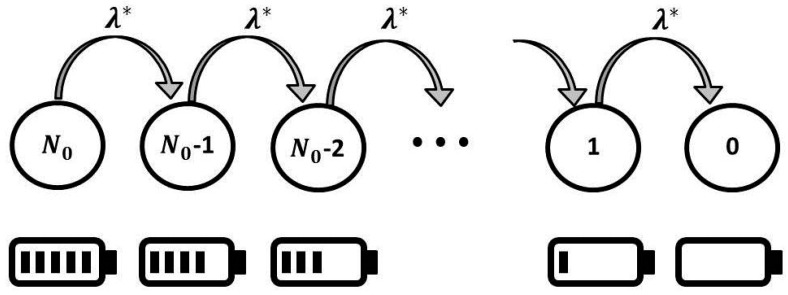
State diagram with absorbing state for sensor battery behavior.

Here, $\lambda_{A}$ is the redundant packet rate, and $p_{A}$ is the probability of the presence of an attack. The state diagram for sensor battery behavior is shown in Figure 2. If $p_{A}=0$, then an attack is absent, and we get the situation described above. If $p_{A}=1$, then the sensor is permanently under attack.

Generally $0<p_{A}<1$ for the following reasons. The intrusion duration can be non-continuous and random. It can be designed by an attacker, in that this attack is energy efficient and hardly detectable. Examples include selective forwarding, random or reactive jamming. In the DoB attack, the set of target sensors and the duration of spoofed packets injection can be randomly defined and dynamically changed, as well. Moreover, the DoB attack can be suspended for reasons beyond the attacker’s control. For example, a sensor can switch itself to sleep mode. The spoofed packets can be recognized and eliminated from the outgoing flow. A sensor does not receive packets if there is buffer overflow, and so on.

Being that the energy consumption for transmitting one packet is not changed, the total number of packets transmitted by a sensor is given by formula (1). The expected sensor lifetime becomes:

(2) $$\mathrm{MTTF}=\frac{N_{0}}{\lambda^{*}},$$

and

(3) $$\mathrm{SSF}=ℋ\left(h,\lambda^{*},N_{0}\right).$$

The result of DoB is $\lambda^{*}/\lambda$ times degradation in MTTF, and:

$$\frac{\lambda^{*}}{\lambda }=1+\frac{\lambda_{A}}{\lambda }p_{A}.$$

To estimate the value of $p_{A}$, an additional model is required. Model details vary, depending on the objectives and scope of the researchers. Here, we consider the particular case as follows. Assume a sensor has the following states:

- Norm—an attack is temporally absent and the sensor functions correctly
- Attack—besides legal packets, a sensor receives and transmits spoofed packets
- Safety—a sensor does not transmit any packets

A sensor enters the Safety state not only under the influence of attack, but also under normal conditions. Generally, firmware of sensors supports sleep mode. It is a widely used approach for energy consumption optimization. Therefore, a sensor can switch to safety mode even if an attack is absent.

In this scenario:

- an attacker attempts to deplete the sensor battery as soon as possible, so the DoB attack can be interrupted if and only if the sensor is switched to the safety mode; and
- safety mode control does not depend on the previous sensor state

**Figure 3:**
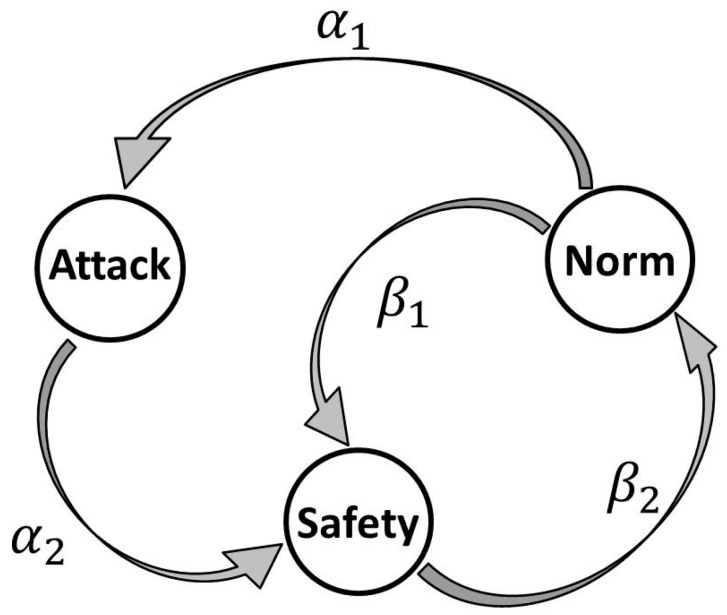
State diagram of sensor modes.

It has been suggested to model sensor functioning by the irreducible aperiodic continuous-time Markov chain, with the states space *Ω* = {Norm, Attack, Safety}, as shown in Figure 3. The presented model is a modification of models from elsewhere [18,53,54].

The infinitesimal generator *G* of the corresponding Markov process is as follows:

$$G=\left(\begin{array}{ccc} -\left(\alpha_{1}+\beta_{1}\right) & \alpha_{1} & \beta_{1}\\ 0 & -\alpha_{2} & \alpha_{2}\\ \beta_{2} & 0 & -\beta_{2} \end{array}\right),$$

where $\alpha_{1}$ is the transition rate to the attack state, and $\alpha_{2}$ is the transition rate from the attack state. These parameters describe the attack intensity and the sensor’s ability to counteract the intrusion. The variable $\beta_{1}$ denotes the transition rate from the normal state to the inactive safety state, while $\beta_{2}$ denotes the transition rate from the safety state to the normal state, when the sensor transmits packets.

Let $p_{N},p_{A},p_{S}$ denote the corresponding equilibrium probabilities, i.e., the vector $\vec{p}=\left(p_{N},p_{A},p_{S}\right)$ satisfies the balance equations:

$$\vec{p}G=0,$$

and

$$\underset{j\in \Omega }{\sum }p_{j}=1.$$

From here, the equilibrium probabilities can easily be derived in closed form. We get:

$$p_{A}=\rho \left(G\right),\;p_{N}=\frac{\alpha_{2}}{\alpha_{1}}\rho \left(G\right),\;p_{s}=\frac{\alpha_{2}}{\alpha_{1}}\;\frac{\alpha_{1}+\beta_{1}}{\beta_{2}}\rho \left(G\right),$$

where

$$\rho \left(G\right)={\left(1+\frac{\alpha_{2}}{\alpha_{1}}+\frac{\alpha_{2}}{\beta_{2}}+\frac{\alpha_{2}\beta_{1}}{\alpha_{1}\beta_{2}}\right)}^{-1}.$$

Therefore, in the considered scenario, we get:

$$\mathrm{MTTF}=\frac{N_{0}}{\lambda_{A}\rho \left(G\right)+\lambda },$$

and

$$\mathrm{SSF}=ℋ\left(h,\lambda_{A}\rho \left(G\right)+\lambda ,N_{0}\right).$$

From the obtained results, it can be concluded that for the same duty cycle rate, it is advisable to increase the sleep mode duration in order to reduce the influence of DoB.

### 4.3. DoB based on Excessive Transmission Power

In this case, an intruder does not use additional packets. However, the radio transceivers of critical sensors are forced to increase transmission power. Assume that in the normal state, a sensor passes packets to a sink through a set of intermediate sensors, $I_{S}$. Under a DoB attack, this sensor transmits packets directly to the sink at a longer distance. In this case, packet transmission rate λ is not changed. However, the energy consumption for transmitting one packet is increased. Let $e_{A}$ be the energy amount consumed for one packet transmissioin under a DoB attack, $e_{A}\gg e_{0}$. Now, the total number of packets transmitted by a sensor, $n_{A}$, can be estimated as follows:

$$n_{A}=C/e_{A}.$$

The sensor battery behavior shown in Figure 2 is the same, except for the number of states, which is drastically reduced: $n_{A}\ll N_{0}.$ Therefore, in this scenario:

(4) $$\mathrm{MTTF}=\frac{n_{A}}{\lambda },$$

and

(5) $$\mathrm{SSF}=ℋ\left(h,\lambda ,n_{A}\right).$$

**Figure 4:**
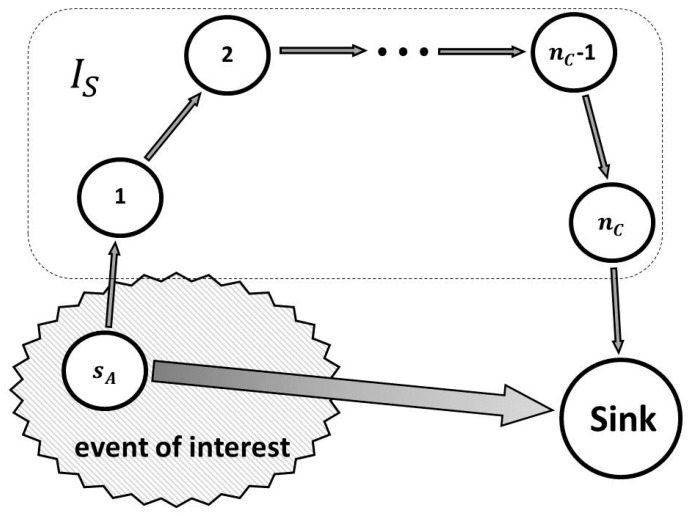
DoB attack based on excessive transmission power.

It is easy to see that the MTTF of the attacked sensor degrades $N_{0}/n_{A}$ times. At the same time the system QoS can be improved. To illustrate this, we consider part of a wireless sensor network, shown in Figure 4.

The sensor $s_{A}$ monitors an event of interest, generates a report, and transmits the report to the sink node through sensors set $I_{S}$. Here, set $I_{S}$ is a chain of $n_{C}$ sensors.

The delivery latency in packets generated by sensor $s_{A}$ is random variable $t_{L}$ and:

$$t_{L}=\overset{n_{c}}{\underset{j=1}{\sum }}t_{j}+t_{0},$$

where $t_{j}$ is the packet service time at node j. It includes time expenses for packet receving, data treatment, waiting, and packet transmitting. The variable $t_{0}$ is the interaction time between the sink and adjacent nodes. The variables $t_{j},$ where $j\in I_{S}$, are assumed to be identically distributed. Therefore, the average delivery latency is:

$$Et_{L}=n_{c}\bar{t}+Et_{0},$$

where $\bar{t}$ is the average service time at a node.

When under DoB attack, sensor $s_{A}$ sends data directly to the sink. Therefore, the average delivery latency becomes $Et_{0}$.

Generally, $\bar{t}\gg Et_{0}$. However, even if $\bar{t}\approx Et_{0}$, the packet delivery latency, which is an important QoS parameter, is reduced significantly: $n_{c}+1$ times.

If $I_{S}$ also receives packets from sensors other than $s_{A}$, and packets losses are possible, then DoB decreases the offered load for $I_{S}$. Therefore, the loss rate is decreased as well. Thus, the system QoS can be improved under DoB. At the same time, system lifetime is essentially degraded. 

The harm from DoB attacks based on excessive transmission power can be stronger, compared to DoB attacks caused by excessive packets, even if not interrupted ($p_{A}=1$). To confirm this proposition, consider MTTF:

$$\frac{n_{A}}{\lambda }<\frac{N_{0}}{\lambda_{A}+\lambda }.$$

Taking into account equality (1), we get:

$$e_{A}>e_{0}\left(1+\frac{\lambda_{A}}{\lambda }\right).$$

If the above inequality is satisfied, then DoB accompanied by QoS improvement is more destructive than powerful DoB attacks accompanied by QoS degradation.

## 5. DoB Detection

In this section, we discuss some aspects of DoB detection. Implementation details of intrusion detection system (as well as specific intrusion prevention and mitigation technique, countermeasures against compromised nodes) are out of scope of this paper.

A defense scheme against intrusions in WSNs can include identification of attack source, localization of victim sensors, activating safe operating mode, and malicious node isolation. But before applying reactive protection, the DoB attack has to be detected. If reactive protection is used in the normal stage, then sensor throughput degrades. Therefore, reactive protection has to be activated in the attack stage and deactivated in the normal stage. Hence, it is important to detect intrusions with high confidence, that is to say, provide a satisfactory level of false positives and false negatives. In the case of a DoS (or DDoS) attack, the principle of collaborative and distributed detection can essentially improve the efficiency of intrusion detection systems [55]. If nodes detect an attack with inconclusive evidence, then a cooperative intrusion detection procedure aggregates the node reports, and provides a decision about the presence of an intrusion. Due to the increment of QoS degradation under DoS, it allows one to recognize attacks in the early stages. The complexity of the DoB detection problem is that the principle of collaborative and distributed detection does not work well in this case. To illustrate, consider the situation in Figure 4.

In the normal state, the energy consumption of sensor $s_{A}$ and the sensors of $I_{S}$ is as follows:

$$E_{N}=\overset{n_{c}}{\underset{j=1}{\sum }}e\left(j\right)+e\left(s_{A}\right).$$

Here and below, e(x) is the energy consumption of sensor *x*. Without loss of generality, assume that adjacent nodes are separated by distance *d*. Taking into account our model assumptions, we get:

(6) $$\forall j\in I_{S}\;e\left(j\right)=e\left(s_{A}\right)=a\cdot d^{\gamma },$$

where γ is called the path loss exponent, and a is a constant defined by the transmitter power amplifier [56]. Therefore:

$$E_{N}=(n_{c}+1)\cdot a\cdot d^{\gamma },$$

Under a DoB attack, the total energy consumption becomes:

$$E_{A}=a\cdot d_{S}^{\gamma },$$

where $d_{S}$ is the distance between sensor $s_{A}$ and the sink node. It is possible to reduce the total energy consumption under DoB. In fact, we get the following inequality:

$$E_{A}<E_{N},$$

and hence

$$d_{S}<d\cdot \sqrt[{\gamma }]{n_{c}+1}.$$

For example, if $\gamma =2,\;n_{c}=8,\;d_{S}=\;3d$, then the total energy consumption is not changed. At the same time, the system lifetime degrades by nine times. If a decision about a DoB presence is based on observation of all sensors, then the attack is not detected.

Thus, it is reasonable to deploy an intrusion detection system at each node. It consumes some system resources, and leads to some QoS degradation, but it is a reasonable price for supporting system security.

For DoB attack detection, the point-of-change detection technique can be applied [57]. The idea of the intrusion detection method is based on the assumption that DoB attacks lead to relatively abrupt changes in the process of sensor battery depletion compared to energy consumption in the normal case. The detection algorithm is a kind of cumulative sums method (also known as CUSUM). The incoming random values for the algorithm are observations of sensor energy consumption per time units. Let $\xi_{j},\;j>1$ be the sensor energy consumption per a time unit (a few seconds). The time units are not necessary equal. It is reasonable to merge the successive units, when the energy consumption is very low. If an attack is launched, then the observed random sequence, $\left\{\xi_{j}\right\}$ changes own properties. Let $T_{attack}$ be the attack start time (change-point). Let us use the designation $T_{alarm}$ for the moment of time when the decision about attack presence is made and the corresponding alarm signal is generated. Generally, it needs to minimize the difference $T_{alarm}-T_{attack}$ expecting that $T_{alarm}>T_{attack}$. If $T_{alarm}<T_{attack}$ then the false alarm takes place. Due to the stochastic character of observed energy consumption, the false alarm can be generated many times. The reaction for false alarms can essentially reduce the network performance. It needs to reduce the false alarm rate as much as possible. A conventional change-point detection approach is based on the cumulative sum:

$$S_{0}=0,\;S_{i}=\max\left(0,S_{i-1}+\Delta_{i}\right).$$

Here, $\Delta_{i}$ is a function of observations $\xi_{i}$. For example, in some approaches, which have been offered for DDoS attacks detecting, the log-likelihood ratio is taken as the function $\Delta_{i}$. It can be applied for DoB detection as well. The alarm time moment is defined as follows:

$$T_{alarm}=\inf\;\left\{i\geq 1:S_{i}>h\right\},$$

where *h* is some threshold value. This scheme has the disadvantage that the proper threshold *h* is hard to evaluate. Generally, it is recommended to define *h* by simulation to get the tradeoff between efficient detection and false alarm rate.

If the battery capacity is large enough, then the fast discharge of sensor battery within a reasonable time is not a significant detriment. Therefore, if the attack is detected during this time, it is possible to avoid a considerable harm. In this case, the efficient method of point-of-change detection with given lag can be applied [58]. Let the admissible lag equals $T_{lag}$ time units. If the alarm signal is declared at any time after $T_{alarm}$ and before $T_{attack}+T_{lag}$, then the attack is considered to be successfully detected. If $T_{alarm}>T_{attack}+T_{lag}$, then the intrusion is successful. Under the condition of fixed false alarm rate, the attack detection probability has to be maximized, while under the fixed probability of attack detection, the probability of false alarm has to be minimized. The provided formulation of the change-point detection problem addresses the case where the cumulative distribution function for $\xi_{j}$ convolution can be derived. In this case, the optimal threshold value can be calculated.

Let $\tilde{\alpha }$ be the required false alarm rate, *w* be the number of observations within $T_{lag}$, $F_{\xi ,w}$ is CDF of the convolution $\xi_{1}*\xi_{2}*\ldots *\xi_{w}$:

$$F_{\xi ,w}\left(x\right)=\mathbb{P}\left(\overset{w}{\underset{j=1}{\sum }}\xi_{j}\leq x\right),\;x\in \mathbb{R}.$$

Therefore, the optimal threshold is as follows:

$$h_{opt}=F_{\xi ,w}^{-1}\left(1-\tilde{\alpha }\right),$$

where $F_{\xi ,w}^{-1}$ is the inverse function of $F_{\xi ,w}$.

If the alarm time moment is defined as follows:

$$T_{alarm}=\inf\;\left\{i\geq w+1:\;\overset{i}{\underset{j=i-w}{\sum }}\xi_{j}>h_{opt}\right\},$$

then the provided threshold maximizes the probability of attack detection. The choice of a particular discord detection procedure depends largely on practical applications and WSNs environment.

## 6. Performance Analysis

In this section, we provide simulation experiments to illustrate the theoretical results, by which we discuss the impacts of the DoB attack parameters on the network survivability metrics as well. The provided results are expected to offer insights into the enhancement of protection mechanisms.

We first consider the sensor lifetime under DoB caused by both excessive packets and excessive transmission power. We set sensor battery capacity $C=1000\;e_{0}$, and the packet transmission rate in the normal case to λ=1.

Besides the deterministic energy consumption of packet transmission, we consider the cases, when it is a random number. In some previous works the battery charge of a sensor had been described by the normal distribution (see, for example, [59]). Since the energy consumption of packet transmission cannot be negative and is limited from above, we use a truncated normal distribution. Let us introduce the following designation for doubly truncated normal distribution function [60]:

$$\Phi_{T}\left(x|\nu ,\sigma^{2},A,B\right)=\frac{1}{\sqrt{\pi }\;\sigma }e^{-{\left(x-\nu \right)}^{2}/2\sigma^{2}}{\left[\frac{1}{\sqrt{\pi }\;\sigma }\int_{A}^{B}e^{-{\left(t-\nu \right)}^{2}/2\sigma^{2}}dt\right]}^{-1},$$

where *A* and *B* are the lower and upper truncation points, respectively.

The following DoB attack scenarios have been considered.

- Case 1. The attack is caused by excessive packets, so, redundant packets rate $\lambda_{A}=7$ and it is assumed that $p_{A}=1$. The energy consumption for transmitting each packet is not changed, and equals $e_{0}$.
- Case 2. The scenario is the same as in Case 1, except that the energy consumption of packet transmission is not deterministic. It is assumed to be a random variable having the cumulative distribution function $\Phi_{T}\left(x|e_{0},1,0,2e_{0}\right)$.
- Case 3. A victim sensor is forced to increase the transmission range by three times, and in formula (6) γ=2, a=1. The packet transmission rate is not changed: *λ* = 1. So, the attack is caused by excessive transmission power.
- Case 4. This is the same as Case 3, except that the energy consumption of packet transmission is randomly distributed with the cumulative distribution function $\Phi_{T}\left(x|e_{A},1,0,2e_{A}\right)$.

In all four cases, a sample of size 1000 was used to analyze the sensor lifetime degradation.

**Figure 5:**
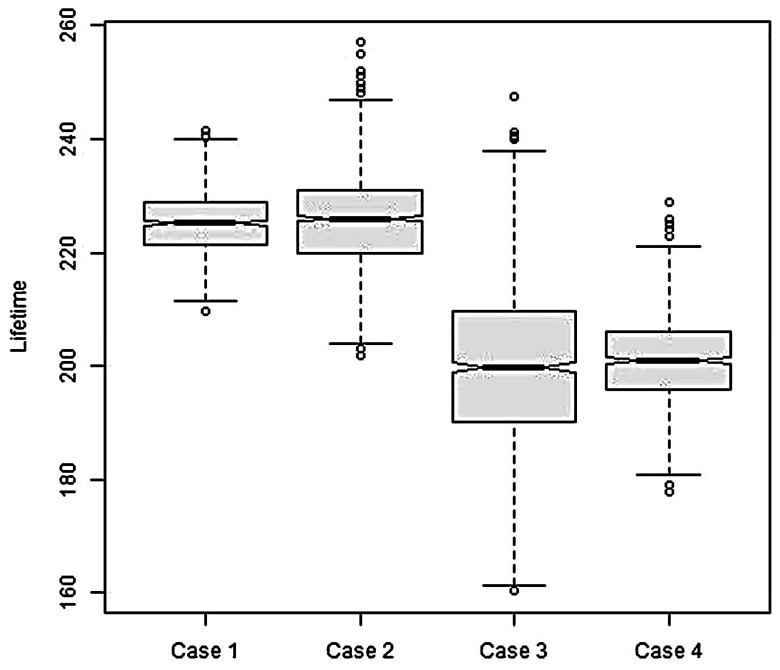
Boxplot diagram for sensor lifetime.

The simulation results correspond to those of analytical expression: Formulas (2) and (3). The cases of deterministic energy consumption of packet transmission are consistent with the cases when this energy consumption has a truncated normal distribution. It is shown in Figure 5.

The bottom and top of the gray boxes are the first and third quartiles. The black line inside each box shows the median. The whiskers stretch from the edge of the boxes to the furthest sample data point that is within 1.5 times the interquartile range. If some points are past the ends of the whiskers, they are displayed with dots. They can be considered to be outliers.

The sample variability in Case 3 is higher than in Case 1. This is not surprising, since the variance of Erlang random variable is directly proportional to the shape parameter, and is inversely proportional to the square of the rate parameter. The theoretically predicted MTTF in Case 1 is 225, and in Case 3 it is 200. It is consistent with the sample-based estimation of MTTF. The detailed statistics is shown in Table 1.

**Figure 6:**
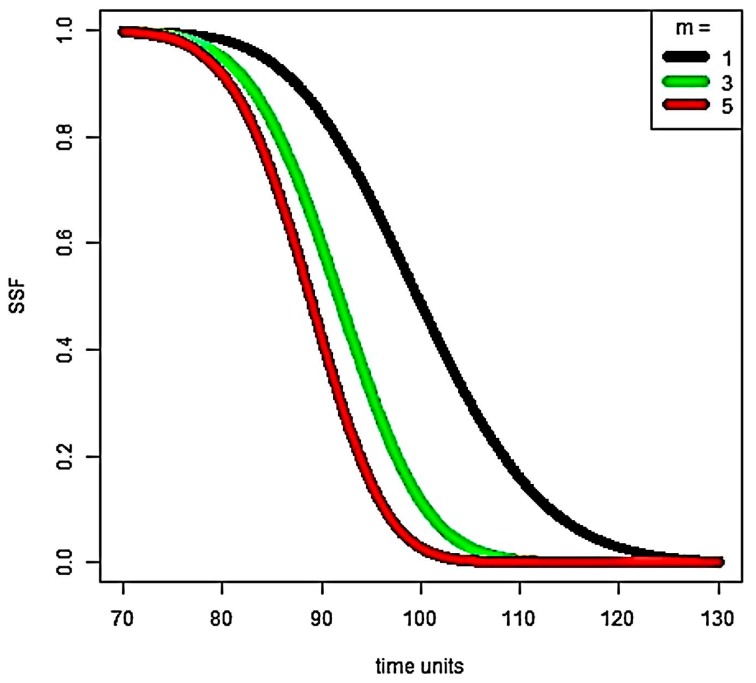
Behavior of the system survivability function, depending on *m*.

We next consider the system survivability function. The behavior of ℋ(h,1100) is shown in Figure 6.

The system survivability is relatively high up to 80% of MTTF, and then it drastically degrades. There is an inverse relationship between the cardinality of the critical sensors set and system survivability; when *m* decreases, SSF increases.

Let us consider two DoB scenarios: an attack caused by spoofed packets, and an attack caused by excessive transmission power. In the first, case the traffic rate is increased ten times, i.e., $\lambda^{*}=10$. In the second case, the energy consumption of packet transmission is increased ten times, as well, $e_{A}=10e_{0}$. All other system parameters are the same. It can be seen that MTTF is the same in both cases.

**Figure 7:**
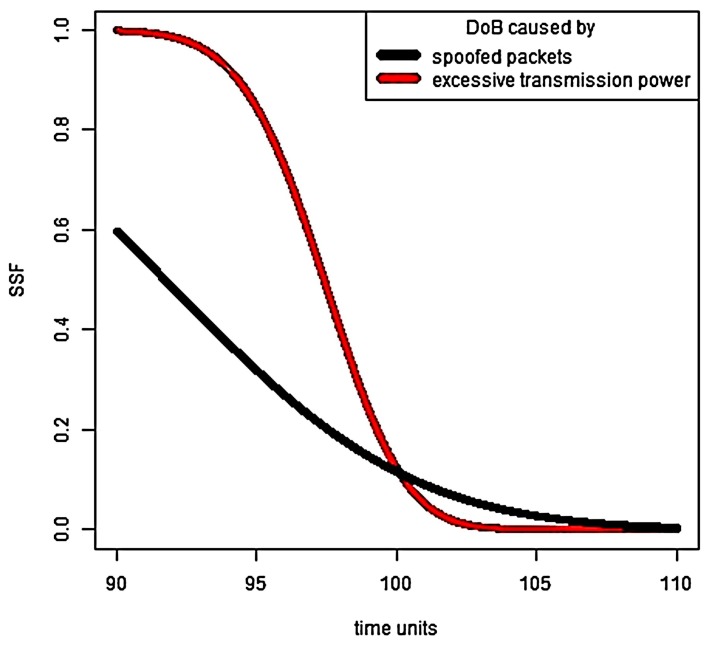
System survivability and varied types of DoB.

The most adversely DoB scenario for the victim sensor depends on time. This is verified through simulation results demonstrated in Figure 7, i.e., the harm from DoB caused by spoofed packets exceeds the harm from DoB caused by transmitting power increasing up to the MTTF point, and after this, we get the inverse situation. Obviously, SSF gets closer and closer to zero as time increases.

We also examine the tradeoff between DoB detection success and false alarm. The system model assumptions are the same as those discussed in Section 3. Under a DoB attack, the energy consumption per time unit is three times higher than the initial value. The false alarm rate takes the values 0.01, 0.05, 0.1, 0.2.

**Figure 8:**
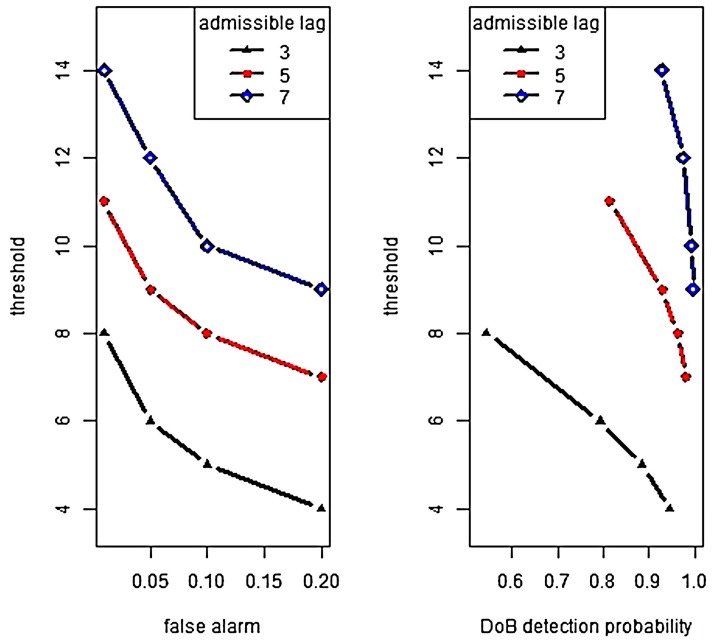
Efficiency of DoB detection.

In Figure 8, the optimal threshold is shown as a function of the false alarm (left plot) and the DoB detection probability (right plot). For example, if the required false alarm rate is 0.05 and $T_{lag}=5$, then the optimal threshold value equals 11. If *h* is larger than the mentioned value, then the required false alarm rate cannot be supported. If *h* < 11, then the false alarm probability is less than 0.05; however, the intrusion detection probability is not minimized. The best detection result under given conditions is achieved with *h* = 11, it is 0.82.

Generally, if the selected threshold exceeds the optimal threshold then the false alarm rate is reduced; however, it reduces the intrusion detection probability as well. From Figure 8, observe that the optimal threshold is an increasing function of $T_{lag}$. This is due to the fact that the change point detection rule is based on sum of non-negative numbers.

Let us remark that the quality of detection algorithms can be essentially improved by increasing admissible lag. If in the example above, the required false alarm rate $\tilde{\alpha }=0.01$ and $T_{lag}=15,$ then the attack is detected with confidence 99.9%. However, it makes sense if the sensor battery capacity is large enough.

## 7. Conclusions

In this paper, we consider a special security threat in sensor networks. The attack is named depletion-of-battery. DoB is considered as a distinguishing type of attack. Their goal is rather a physical harm, while QoS can be temporarily improved. Energy exhausting attacks, which had been considered previously, are accompanied by QoS degradation (e.g., vampire attacks). DoB can be caused by deliberate action or a random combination of circumstances. To estimate the effect of the DoB attack, corresponding mathematical models based on stochastic processes have been developed. The protection approach can be based on independent power control for sensors and also on effective monitoring based on the methods of discord detection. The proposed theoretical framework is supported by simulation results. In our future work, we will develop a comprehensive intrusions taxonomy, which includes the considered DoB attack and their variations. We plan to extend the system survivability analysis into DoB mitigation schemes. For these purposes, we will develop the corresponding system models based on continuous-time continuous-state processes. In addition, as a future research challenge, we envision that the development of intrusion detection systems for various scenarios of WSNs/IoT applications will be a promising research direction.

## Figures and Tables

**Table 1:** Detailed statistics for sensor lifetime. *Min* is the smallest value in the sample. *Max* is the largest value in the sample. *Mean* is the sample mean. *Median* is the second quartile. *Q* 1 and *Q* 3 are the first and third quartiles.

Scenario	*Min*	*Q* 1	*Median*	*Mean*	*Q* 3	*Max*
**Case 1**	209.6	221.4	225.2	225.2	228.8	241.5
**Case 2**	202.0	220.0	226.0	225.8	231.0	257.0
**Case 3**	160.4	190.2	199.8	200.2	209.6	247.6
**Case 4**	178.0	196.0	201.0	200.8	206.0	229.0
